# EFFECT OF OLDER ADULT HEALTH STATUS AND AGE ON TRAINEES’ PERCEPTIONS OF CLINICAL WORK WITH OLDER ADULT CLIENTS

**DOI:** 10.1093/geroni/igad104.1030

**Published:** 2023-12-21

**Authors:** Grace Caskie, Benjamin Johnson, Eve Root

**Affiliations:** Lehigh University, Bethlehem, Pennsylvania, United States; Lehigh University, Bethlehem, Pennsylvania, United States; Lehigh University, Bethlehem, Pennsylvania, United States

## Abstract

The growing older adult population in the US will result in more psychologists providing services to older clients. Yet, most psychology doctoral programs lack specific training on mental healthcare for older adults, which allows ageism and healthism to remain unchallenged. To examine how age and health status affect trainees’ clinical perceptions, we used a 2 age X 3 health repeated-measures experimental design. Doctoral trainees (N=219; age=21-58) in clinical and counseling psychology were randomly assigned to the 71-year-old or 81-year-old condition. Trainees then read three randomly-ordered clinical vignettes depicting clients with depressive symptoms that varied health condition (healthy, recent Alzheimer’s diagnosis, heart disease). Trainees provided clinical ratings for each client. Regardless of age, relative to the client with a recent Alzheimer’s diagnosis, the healthy client was perceived as more likely to develop an effective therapeutic relationship, and both the healthy client and client with heart disease were considered more appropriate candidates for therapy. For the 71-year-old client, prognosis, competence, and comfort did not differ between the healthy client and the client with heart disease, but both were rated significantly better than the client with Alzheimer’s. For the 81-year-old client, competence and comfort treating were significantly lower for the client with Alzheimer’s as compared to both the client with heart disease and the healthy client, but trainees also expressed less comfort and competence treating the client with heart disease than the healthy client. Doctoral psychology programs should include training addressing trainees’ clinical knowledge and biases to ensure competent care for older adults.

